# Identification and validation of circulating miRNAs as endogenous controls in obstructive sleep apnea

**DOI:** 10.1371/journal.pone.0213622

**Published:** 2019-03-13

**Authors:** Fernando Santamaria-Martos, Ivan Benítez, Andrea Zapater, Cristina Girón, Lucía Pinilla, Jose Manuel Fernandez-Real, Ferran Barbé, Francisco Jose Ortega, Manuel Sánchez-de-la-Torre

**Affiliations:** 1 Group of Translational Research in Respiratory Medicine, Hospital Universitari Arnau de Vilanova y Santa Maria, IRB Lleida, Lleida, Spain; 2 Centro de Investigación Biomédica en Red de Enfermedades Respiratorias (CIBERES), Madrid, Spain; 3 Department of Diabetes, Endocrinology and Nutrition, Institut d'Investigació Biomèdica de Girona (IdIBGi), Girona, Spain; 4 CIBER de la Fisiopatología de la Obesidad y la Nutrición (CB06/03) and Instituto de Salud Carlos III, Madrid, Spain; Chuo University, JAPAN

## Abstract

microRNAs (miRNAs) are non-coding RNAs highly relevant as biomarkers for disease. A seminal study that explored the role of miRNAs in obstructive sleep apnea syndrome (OSA) demonstrated their usefulness in clinical management. Nevertheless, the miRNAs that may act as endogenous controls (ECs) have not yet been established. The identification of ECs would contribute to the standardization of these biomarkers in OSA. The objective of the study is to identify miRNAs that can be used as ECs in OSA. We evaluated 100 patients divided into two different cohorts: a learning cohort of 10 non-OSA and 30 OSA patients, and a validation cohort (20 non-OSA and 40 OSA patients). In the learning cohort, a profile of 188 miRNAs was determined in plasma by TaqMan Low Density Array. The best EC candidates were identified by mean center+SD normalization and concordance correlation restricted normalization. The results were validated using NormFinder and geNorm to assess the stability of those ECs. Eight miRNAs were identified as EC candidates. The combination miRNA-106a/miRNA-186 was identified as the most stable among all candidates. We identified a set of ECs to be used in the determination of circulating miRNA in OSA that may contribute to the homogeneity of results.

## Introduction

Obstructive sleep apnea (OSA) is a prevalent disease that affects approximately 10% of adults [1–3]. It is caused by intermittent collapse of the upper airway during sleep, which leads to oxygen desaturation, arousals and intrathoracic pressure changes. OSA is associated with hypertension, cardiovascular and cerebrovascular diseases, diurnal somnolence and decreased quality of life [4].

Understanding the causes and consequences of OSA and identifying actions to perform personalized medicine are research priorities in the field [5,6]. One of the greatest challenges is identifying accurate and feasible biomarkers useful in diagnosis, prognosis and response to treatment. This challenge has stimulated an interest in novel biomarkers to improve OSA diagnosis and comprehensive management [7], through which micro-ribonucleic acids (miRNAs) have emerged as a new opportunity [8].

microRNAs (miRNAs) are small non-coding RNA molecules that represent an important class of regulatory epigenetic mechanisms [9]. miRNAs influence important cellular functions, such as development and differentiation, and play a pivotal role in many biological processes related to health and disease [10–12]. miRNAs can be rapidly released from tissue into circulation in several pathologies and exist in a high-stability, cell-free form in plasma [13]. Therefore, circulating miRNAs have great potential as non-invasive biomarkers for molecular diagnostics and prognostics and as therapeutic targets [14].

The role of miRNAs in OSA has been little studied. A seminal study demonstrated the utility of these molecules as biomarkers of adequate response to the treatment of OSA with continuous positive airway pressure (CPAP) [8]. However, prior to any analysis of circulating miRNAs, normalization is crucial due to the variability that occurs in plasma RNA isolation and analysis [14,15].

Normalization is a process aimed at differentiating biological variation from experimental artefacts. Normalization reduces the variability due to technical error and contributes to standardization and homogeneity in the analysis of miRNAs [16]. Currently, mean-center normalization is the most accurate method to normalize the results when a high number of miRNAs are being analysed [17]. However, a small number of miRNAs are often analysed, in which case mean-center normalization is not a valid method; instead, endogenous control (EC) normalization is the best option [14,17]. Therefore, suitable ECs (i.e., those that are disease- and tissue-specific, highly detected in all samples, and stable both within and between groups) are needed. The use of unsuitable normalizers can lead to misleading results [18–20].

To the best of our knowledge, there is no current consensus on EC miRNAs for quantitative polymerase chain reaction (qPCR) analysis of plasma miRNAs in OSA. Therefore, the aim of this study was to identify suitable ECs for normalization of circulating miRNAs in OSA studies with the objective of standardizing the analysis of these biomarkers in OSA.

## Methods

### Study cohort and sample collection

**Fig 1:**
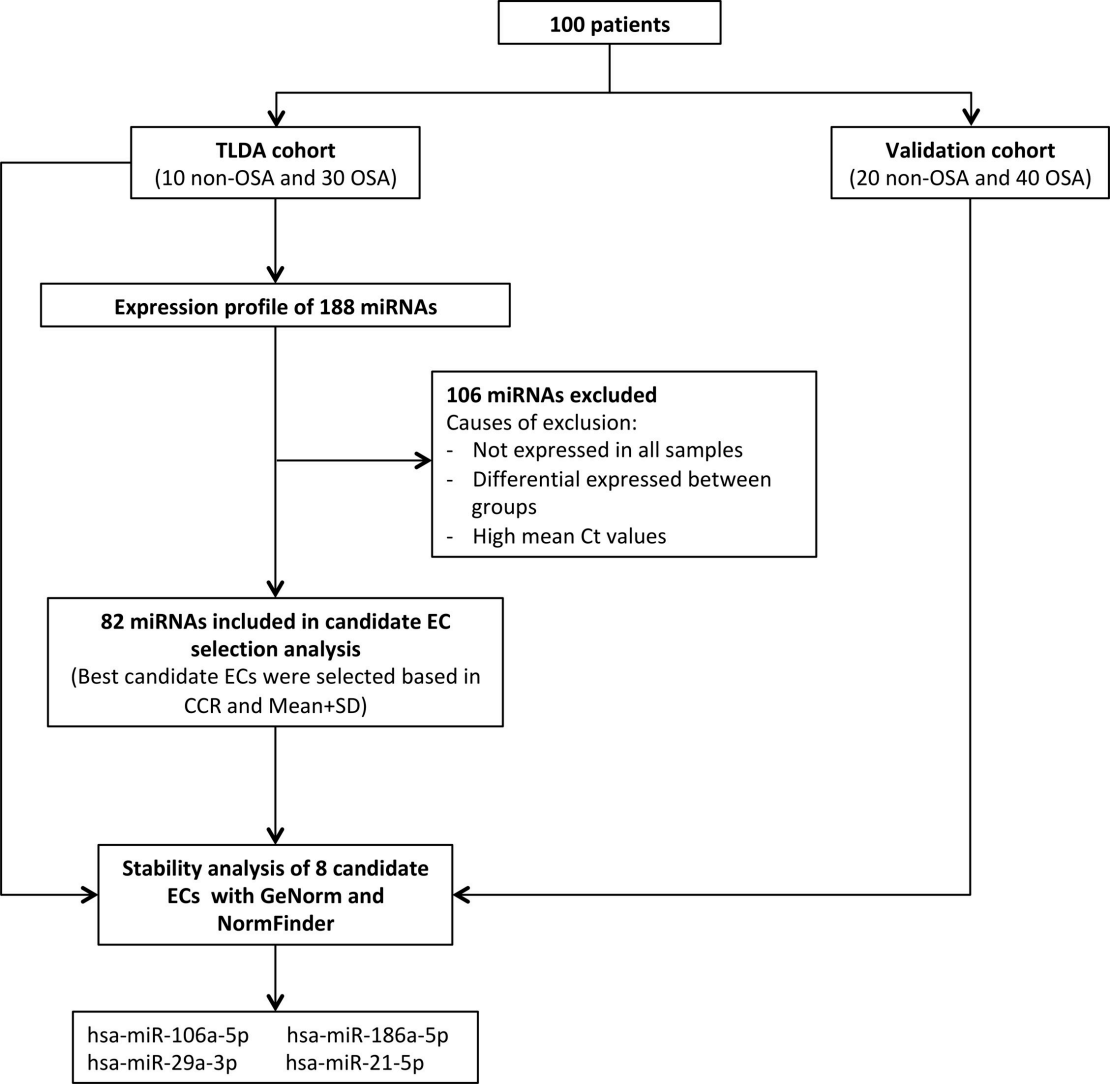
Flowchart of the study. Patients who were referred because of suspected OSA were divided into the TLDA cohort (general profile of miRNAs) and the validation cohort and further divided for study on the basis of non-OSA and OSA. Ten non-OSA and 30 OSA patients were used as the TLDA cohort. miRNAs that do not follow the characteristics of a good EC were excluded. Two different strategies (mean-center+SD and CCR) were used in order to select candidate ECs. Stability analysis by GeNorm and NorFinder was performed in the TLDA cohort and in an independent validation cohort (20 non-OSA and 40 OSA patients).

One hundred patients aged between 18 and 60 years and referred because of suspected OSA were enrolled at the Sleep Unit of Santa Maria Hospital (Lleida, Spain). OSA was diagnosed via a conventional polysomnographic sleep study. The results from all sleep studies were analysed by trained personnel, using standard criteria. Apnea was defined as an interruption or reduction of oronasal airflow ≥ 90% for at least 10 seconds. Hypopnea was defined as a 30–90% reduction in oronasal airflow for at least 10 seconds associated with an oxygen desaturation of at least 3% or an arousal on the electroencephalogram. The apnea-hypopnea index (AHI) was calculated based on the average number of apnea plus hypopnea episodes per hour of sleep. The cohort was split into two cohorts: i) TaqMan Low Density Array (TLDA) cohort: an initial sample of 40 male patients (10 patients without OSA and 30 patients with OSA) matched by age and body mass index (BMI). A general miRNA profile was performed. ii) Validation cohort: the remaining 60 patients (20 patients without OSA and 40 with OSA) were used to validate the candidate ECs (Fig 1). The initial exclusion criteria discarded patients with previous use of CPAP and any condition that, in the opinion of the responsible physician investigator, made the person unsuitable for the study (e.g., pregnancy, drug and alcohol consumption or less than one year of life expectancy). TLDA cohort patients were selected who had no other comorbidities apart from those typically associated with OSA (e.g., hypertension, dyslipidaemia, cardiovascular events). All recruited patients signed an informed consent form in accordance with the Helsinki Declaration of 1964 and the ethics committee of the centre (Clinical Research Ethics Committee (CEIC) of the Arnau de Vilanova University Hospital) approved the study. All methods were performed in accordance with current clinical practice guidelines and regulations. A venous fasting blood sample was obtained from each patient between 08.00 and 09.00 a.m. Plasma was obtained by standard venepuncture and centrifugation in EDTA-coated tubes (Vacuette, Greiner Bio-One, Kremsmünster, Austria). Plasma was separated via centrifugation at 1500 g for 10 min at 4°C. All specimens were immediately aliquoted, frozen, and stored in a dedicated -80°C freezer. No freeze-thaw cycles were performed during the experiment.

### Circulating RNA extraction and purification

RNA extraction was performed from 300 μL of plasma using a mirVana PARIS isolation kit (Applied Biosystems, Vilnius, Lithuania) according to the manufacturer’s instructions. Non-human cel-miR-39 was spiked into the plasma immediately before extraction. RNA concentration and integrity were examined by RT-qPCR quantification of cel-miR-39. RNU6 was selected as the plasma quality indicator because it is highly expressed in different cell types but not well detected in plasma. High detection of RNU6 in plasma indicates cellular contamination [19].

### TaqMan low density array

A general profile with 188 miRNAs reported in previous experiments as the most prevalent in plasma were selected for profiling [21–23]. qRT-PCR was performed on the RNA of 40 patients using TaqMan Low Density Array (TLDA) human microRNA custom TLDAs (Life Technologies, Foster City, CA, USA). Briefly, a fixed volume of 3 μL of RNA solution from the 40 μL of RNA isolation eluate was used as the input for retrotranscription using a TaqMan MicroRNA Reverse Transcription Kit and the TaqMan MicroRNA Multiplex RT Assays, which are customised to run the TLDAs. Preamplification was performed using TaqMan PreAmp Master Mix and Megaplex PreAmp Primers for our custom selection. RT-PCR was carried out by means of an Applied Biosystems 7900HT thermocycler.

### Candidate EC selection

ECs are miRNAs that are highly represented in all study samples and show similar abundances between groups. Two different approaches were used to choose the best candidates in OSA: i) the miRNA with the values most similar to the global mean expression [24], which was determined by normalizing the data set with the global mean to select the miRNA with the smallest standard deviation (mean-center+SD); and ii) concordance correlation restricted (CCR) normalization procedures, which use a concordance correlation coefficient to select miRNAs that are concordant with the global mean of the fully detected miRNAs [17]. Numerous strategies have been proposed to select the best EC from miRNA arrays [25]. The latest proposals indicate that the similarity between the values of an EC and the global mean (gold standard) is one of the best approximations. Based on these studies, we decided to select the five miRNAs with the lowest variability after normalization by the global mean [23] (mean center+SD) and the five with the highest concordance correlation coefficient of agreement with the global mean of the miRNAs detected in all samples [16] (CCR) as candidates for EC.

### RT-PCR validation

Two-step validation was performed. First, the miRNAs selected as candidate ECs were validated internally to check the variability of determination when changing from an RT-PCR miRNA array to individual RT-PCR. Then, external validation of the EC candidates was performed with an external cohort using individual miRNA TaqMan hydrolysis probes.

### Candidate EC stability analysis

To determine the miRNAs with the best properties to be ECs, we estimated the stability of the candidate ECs. Stability of each candidate EC was performed using the geNorm [26] and NormFinder [27] algorithms. The ranking resulting from each method and its concordance were studied. Furthermore, we evaluated the best combination with the NormFinder algorithm based on the pair that reduced variability the most.

All the analyses were performed using R-project version 3.3.1 (R Foundation for Statistical Computing, Vienna, Austria).

## Results

### Patient characteristics

The 100 patients were middle-aged (less than 60 years old), overweight-obese and mainly males. The clinical and demographic characteristics of the patients are shown in Table 1.

**Table 1:** Baseline characteristics of the patients.

		TLDA cohort			Validation cohort		
		Non-OSA (AHI<15 events/h)	OSA (AHI≥15 events/h)	p-value	Non-OSA (AHI<15 events/h)	OSA (AHI≥15 events/h)	p-value
		N = 10	N = 30		N = 20	N = 40	
Gender: Male n (%)		10 (100%)	30 (100%)		12 (60.0%)	30 (75.0%)	0.37
Age (years), median [IQR]		48.5 [41.8;55.5]	52.0 [44.2;55.8]	0.743	46.0 [41.5;50.2]	51.0 [43.8;55.2]	0.033
BMI (kg/m^2^), median [IQR]		26.1 [23.3;26.9]	27.1 [25.9;30.0]	0.089	29.8 [26.2;36.3]	33.1 [28.7;36.0]	0.279
Hip perimeter (cm), mean (SD)		94.6 (10.8)	101 (8.44)	0.104	106 (15.5)	109 (13.1)	0.391
Waist perimeter (cm), median [IQR]		102 [98.0;104]	104 [100;108]	0.301	111 [102;118]	111 [106;118]	0.849
Physical activity n (%)				0.385			0.356
	Sedentary	4 (40%)	15 (50%)		9 (45.0%)	17 (43.6%)	
	Moderate	3 (30%)	12 (40%)		6 (30.0%)	17 (43.6%)	
	Active	3 (30%)	3 (10%)		5 (25.0%)	5 (12.8%)	
AHI (events/h), median [IQR]		8.45 [6.18;10.8]	32.3 [27.7;49.6]	<0.001	7.52 [4.99;9.01]	38.9 [24.8;70.0]	<0.001
TSat90 (%), median [IQR]		0.08 [0.00;0.14]	2.34 [0.31;6.90]	<0.001	0.12 [0.00;0.32]	4.23 [1.73;18.8]	<0.001
TSat90 (%), median [IQR]		0.08 [0.00;0.14]	2.34 [0.31;6.90]	<0.001	0.12 [0.00;0.32]	4.23 [1.73;18.8]	<0.001

IQR: interquartile range; SD: standard deviation; BMI: body mass index; AHI: apnea-hypopnea index (number of events·h-1); TSat90: percentage of time spent with oxygen saturation less than 90%.

### Selection of the most suitable EC candidates by RT-PCR miRNA array

**Fig 2:**
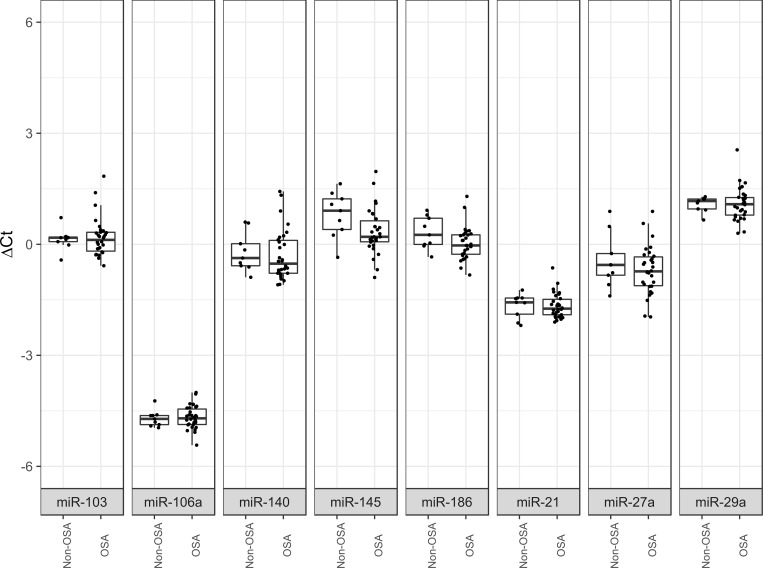
Boxplot comparing OSA and control Ct values. The eight miRNAs were highly detected, and non-significant differences were found between groups.

Among the general profiles, 8 candidate ECs were selected based on the two different methods (Table 2). The eight EC candidates exhibited the desirable characteristics of a good normalizer in that they were highly detected in all samples and were not significantly different between the OSA and non-OSA patients (Fig 2).

**Table 2:** Candidate ECs.

MiRNA Name	Molecule type	Accesion Number*	Mature Sequence	Selection method
**hsa-miR-103a-3p**	miRNA	MIMAT0000101	AGCAGCAUUGUACAGGGCUAUGA	CCR and MC+SD
**hsa-miR-106a-5p**	miRNA	MIMAT0000103	AAAAGUGCUUACAGUGCAGGUAG	MC+SD
**hsa-miR-140-3p**	miRNA	MIMAT0004597	UACCACAGGGUAGAACCACGG	CCR
**hsa-miR-145-5p**	miRNA	MIMAT0000437	GUCCAGUUUUCCCAGGAAUCCCU	CCR
**hsa-miR-186-5p**	miRNA	MIMAT0000456	CAAAGAAUUCUCCUUUUGGGCU	CCR and MC+SD
**hsa-miR-21-5p**	miRNA	MIMAT0000076	UAGCUUAUCAGACUGAUGUUGA	MC+SD
**hsa-miR-27a-3p**	miRNA	MIMAT0000084	UUCACAGUGGCUAAGUUCCGC	CRR
**hsa-miR-29a-3p**	miRNA	MIMAT0000086	UAGCACCAUCUGAAAUCGGUUA	MC+SD
**cel-miR-39-3p**	miRNA	MIMAT0000010	UCACCGGGUGUAAAUCAGCUUG	Spike-in control
**RNU6**	snoRNA	NR_004394**	GTGCTCGCTTCGGCAGCACATATACTAAAATTGGAACGATACAGAGAAGATTAGCATGGCCCCTGCGCAAGGATGACACGCAAATTCGTGAAGCGTTCCATATTTT	Plasma quality indicator

*mirBase database accession number

**NCBI Gene ID

To identify the most stable candidate ECs, the GeNorm and NormFinder algorithms were applied. Both methods reported miR-106a and miR-29a as the most stable. In contrast, miR-27a and miR-145 were the most variable (Table 3). Moreover, NormFinder identified miR-106a and miR-186 as the combination that best reduced the variability among profiles. To verify the ECs’ stability in other OSA classifications, patients were also divided into non-OSA, moderate OSA and severe OSA groups. A similar analysis was performed, and miR-106a/miR-186 remained the most stable pair, followed by miR-29a and miR21 (data not shown).

**Table 3:** Stability ranking of ECs.

	TaqMan Low Density Array				External validation			
	GeNorm		NormFinder		GeNorm		NormFinder	
	Ranking	Stability value*	Ranking	Stability value	Ranking	Stability value	Ranking	Stability value
1	miR-106a	0.500	miR-106a	0.108	miR-186	0.656	miR-186	0.051
2	miR-29a	0.590	miR-29a	0.121	miR-106a	0.686	miR-21	0.071
3	miR-103	0.600	miR-186	0.126	miR-21	0.695	miR-106a	0.072
4	miR-186	0.602	miR-21	0.127	miR-29a	0.716	miR-29a	0.080
5	miR-21	0.611	miR-140	0.139	miR-140	0.757	miR-140	0.107
6	miR-140	0.704	miR-103	0.144	miR-27a	0.782	miR-27a	0.109
7	miR-27a	0.734	miR-145	0.180	miR-103	0.882	miR-103	0.137
8	miR-145	0.751	miR-27a	0.187	miR-145	1.078	miR-145	0.168
Best combination			miR-106a and miR-186				miR-106a and miR-186	

*Stability values are not comparable between methods.

**Fig 3:**
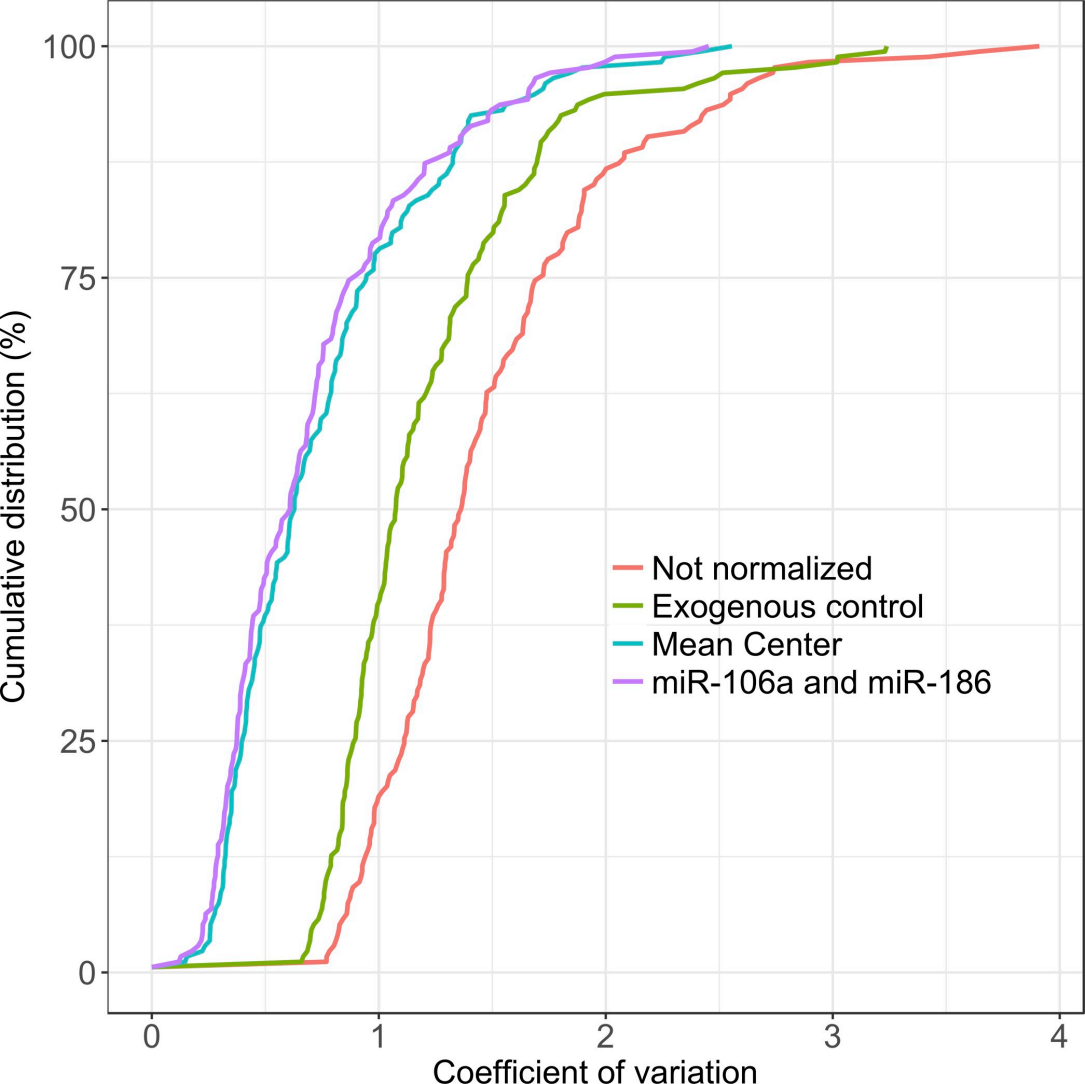
Cumulative distribution plot of the different normalization strategies.

Different strategies of normalization (mean-center, exogenous control and EC selection) were evaluated by comparing their reduction of variability (Fig 3). The variability reduction obtained with mean center (gold standard) was also achieved with EC selection. Exogenous control showed less capacity to reduce variability. A cumulative distribution plot of the coefficient of variation of all miRNA Ct values was examined before and after normalization, confirming the suitability of the ECs as normalizers.

### Internal validation by individual probes

The variability between miRNA arrays and individual probes was evaluated by internal validation in the TLDA cohort. Then, the GeNorm and NormFinder algorithms were used to rank the EC candidates. Similar results were obtained from the TLDA and individual probes (data not shown).

### Confirmation of ECs by individual probes in an external cohort

**Fig 4:**
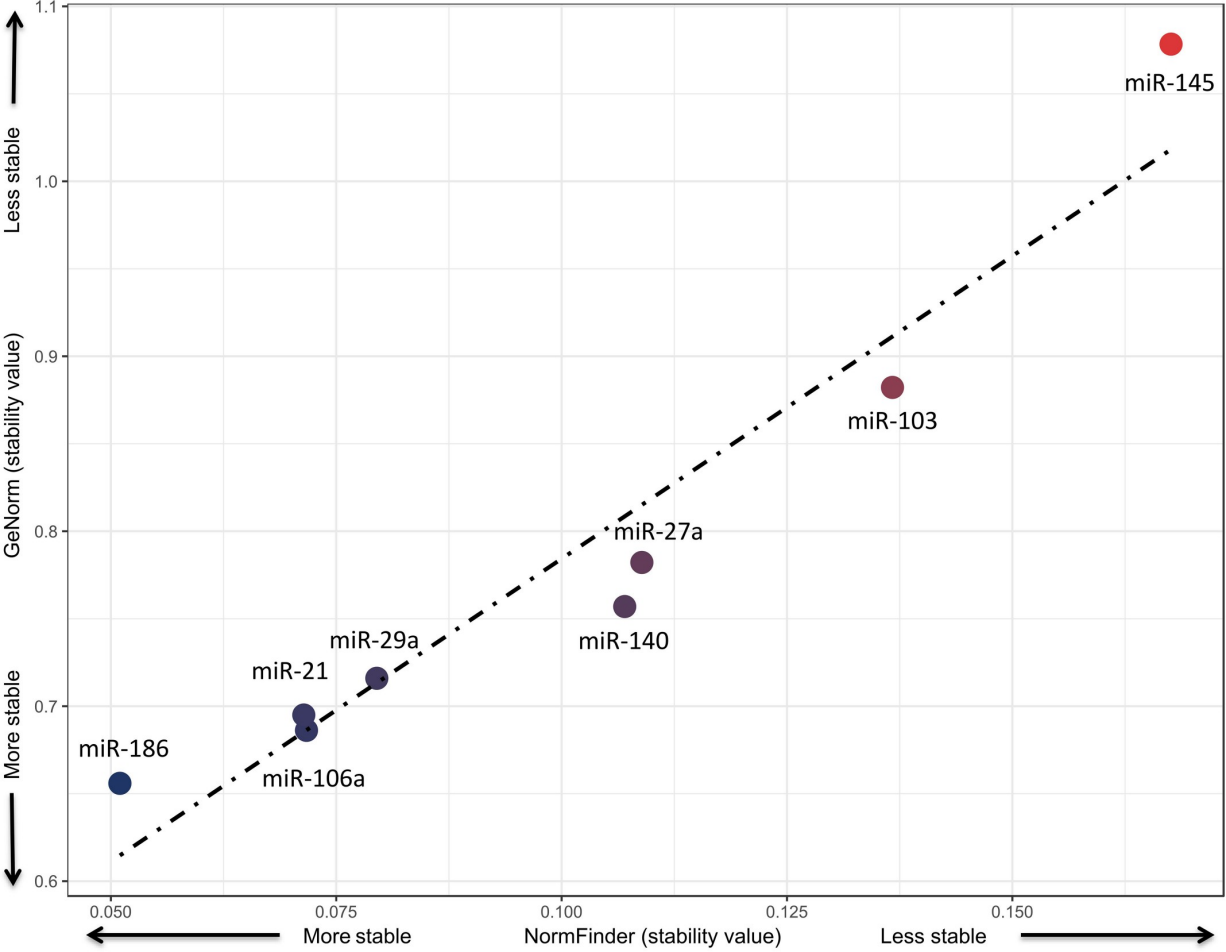
Correlation plot of stability values from NormFinder and GeNorm.

Low Ct values of EC candidates were observed in all patients. Furthermore, when comparing differences between groups, none of the miRNAs showed significant differences. The GeNorm and NormFinder algorithms reported miR-186, miR-106a, miR-21 and miR-29a as the miRNAs with the highest stability (see Table 3 and Fig 4). In contrast, miR-145, miR-103 and miR-27a showed the lowest stability (see Table 3 and Fig 4). Both methods showed high concordance with respect to their stability values. The results showed that miR-186 is the best EC, followed by miR-106a, miR-21 and miR-29, which have similar values of stability for both methods (see Fig 4).

## Discussion

miRNAs are new, reliable, non-invasive biomarkers. Because they play a major role in the development and prognosis of different diseases, their use could go beyond being a simple biomarker. Their utility has been demonstrated in the management of several diseases [28] as well as in the personalized management of OSA [8]. Plasma miRNAs could help in the identification of new OSA phenotypes for personalized management. In the present study, we identified a set of miRNAs that act as ECs for the use of these biomarkers in sleep apnea.

The use of circulating miRNAs represents an advance in the field of minimally invasive biomarkers [29]. Due to their simple and rapid quantification, hundreds of miRNAs can be evaluated simultaneously in several patients, making them one of the most important tools in precision medicine [30].

There are several methods for quantifying miRNAs [31]. Among them, RT-PCR is currently the most commonly available and reliable method. In a couple of hours, this technique allows the levels of miRNAs to be evaluated to perform relative quantification analysis [31,32]. The main problem that currently exists in the relative quantification of miRNAs is technical variability. Currently, there is not a universal method that eliminates such variability. Different techniques have been used, such as spike-in normalization, gene normalization, and small nucleolar RNA normalization, leading to non-replicable results [14,33]. The gold standard—normalization—is based on mean-center methods and is very useful when high-throughput miRNA approximations are being analysed, but it is not an option for the analysis of a few miRNAs. In such cases, the use of ECs is the best option [17,24]. ECs are specific and should be identified for each disease and tissue. The identification and validation of specific suitable ECs is perhaps the most critical step in the analysis of miRNAs to avoid inaccurate interpretation of the data that may lead to biased results. An ideal EC gene will be detected at a constant level across all samples, exhibit relatively stable levels between samples and groups and have no known association with the disease under investigation [16,34]. To the best of our knowledge, this is the first study that aims to identify and validate suitable ECs in OSA.

In the present study, we performed different bioinformatics analyses to determine the most reliable ECs among OSA patients. We evaluated 188 miRNAs and selected eight EC candidates (miR-106a, miR-186, miR-29a, miR-21, miR-103, miR-27a, miR-140 and miR-145), one spike-in synthetic miRNA (cel-miR-39) and one commonly use normalizer (RNU6). Although RNU6 and cel-miR-39 are commonly used in the normalization of miRNAs [14], we used them only as quality indicators. RNU6 is not highly detected in plasma; low Ct values are obtained when haemolyses occurs. Cel-miR-39 has been used only to assess the efficiency of the process, which requires repeating all samples with low efficiency.

The lack of a comprehensive analysis of normalizers for miRNAs in OSA patients could compromise miRNA results. For this reason, we performed miRNA array screening including 188 miRNAs as potential ECs. The data were analysed using mean-center normalization methods to identify the best candidates to serve as ECs. The best normalization strategy for miRNA analysis in OSA patients was identified as the combined use of miR-186 and miR-106a to normalize RT-PCR results. The combined use of 2 or more miRNAs leads to more reliable results than using either one alone. miR-29a and miR-21 are also suitable as ECs for miRNA analysis in OSA.

Different studies previously used miR-186 [35], miR-106a [14,36,37], miR-29a [38] and miR-21 [39] as ECs in other diseases.

The present study has several limitations that deserve comment. First, only patients between 18 and 60 years old have been studied, and larger studies should be performed to determine the validity of these ECs in patients of other ages. Although a large quantity (188) of miRNAs have been studied, not all of them have been analysed; it is possible that other miRNAs would be better ECs, but this does not invalidate the results, because the stability (when compared with other diseases) is sufficiently high. No other comorbidities apart from those typically associated with OSA (e.g., cardiovascular disease, diabetes, and obesity) have been included in the analysis. However, the use of TLDA did reduce the technical variability of the process, in turn providing reliable results. Despite a limited number of non-OSA patients being included in the learning phase (10 non-OSA subjects in the TLDA cohort), the results were validated in a large patient cohort, and the stability values were assessed using two different methods.

## Conclusions

The present study represents the first step in the standardization of the analysis of miRNAs as biomarkers in OSA. In the era of precision medicine, miRNA analysis could open a new field in the search for biomarkers of clinical utility in OSA. These biomarkers could offer insight into the physiopathology of the disease and could have diagnostic and prognostic utility in OSA. The results of this study identified a set of miRNAs that could be used as ECs for standard normalization of miRNA profiling in OSA.

## Supporting information

S1 Dataset Baseline characteristics of TLDA patients. (TXT)

S2 Dataset Baseline characteristics of qPCR patients. (TXT)

S3 Dataset Raw miRNA Ct data from TLDA cohort. (TXT)

S4 Dataset Raw miRNA Ct data from qPCR cohort. (TXT)

S5 Dataset Normalized miRNA Ct from TLDA cohort. (TXT)

S1 Table Normalized miRNA TLDA data (ΔCt). CI: Confidence Interval; IQR: Interquartile Range.
(DOC)
