# Supplementary material for: Identification and validation of circulating miRNAs as endogenous controls in obstructive sleep apnea
Source: PLoS One. 2019 Mar 13;14(3):e0213622. doi: 10.1371/journal.pone.0213622 (PMC6415855; doi:10.1371/journal.pone.0213622)
Supplement: S1 Table — CI: Confidence Interval; IQR: Interquartile Range. (DOC) [file pone.0213622.s006.doc]

**Identification and Validation of Circulating miRNAs as Endogenous Controls in Obstructive Sleep Apnea**

**Running Title:** miRNAs Endogenous Controls in Obstructive Sleep Apnea

**Authors:** F. Santamaria-Martos^1^, I. Benítez^1,2^, A. Zapater^1^, C. Girón^1^, L. Pinilla^1^, J.M. Fernandez-Real^3,4^, F. Barbé^1,2^, F. Ortega^3,4,*^, M. Sánchez-de-la-Torre^1,2,*^.

**Affiliations:**

^1^Group of Translational Research in Respiratory Medicine, Hospital Universitari Arnau de Vilanova y Santa Maria, IRB Lleida, Lleida, Spain;

^2^Centro de Investigación Biomédica en Red de Enfermedades Respiratorias (CIBERES), Madrid, Spain.

^3^Department of Diabetes, Endocrinology and Nutrition, Institut d'Investigació Biomèdica de Girona (IdIBGi), Girona, Spain;

^4^CIBER de la Fisiopatología de la Obesidad y la Nutrición (CB06/03) and Instituto de Salud Carlos III, Madrid, Spain;

*Correspondence to:

Manuel Sánchez-de-la-Torre, PhD, Hospital Arnau de Vilanova-Santa María, IRBLleida, CIBERES, Avda. Rovira Roure 80, 25198, Lleida, Spain; Phone: +34973702959; e-mail: sanchezdelatorre@gmail.com

Francisco Ortega Delgado, PhD, Department of Diabetes, Endocrinology and Nutrition, Institut d'Investigació Biomèdica de Girona (IdIBGi), Dr. Castany s/n, 17190 Salt, Girona, Spain; fortega@idibgi.org

**Keywords:** Obstructive sleep apnea, microRNA, Endogenous controls, profiling, biomarkers.

**S1 Table:**  normalized miRNA TLDA data (ΔCt).

|  |  | Non-OSA | | |  | OSA | |
| --- | --- | --- | --- | --- | --- | --- | --- |
|  |  | *Mean (95%CI)* |  | *Median [IQR]* |  | *Mean (95%CI)* | *Median [IQR]* |
| **miRNA (ΔCt)** |  |  |  |  |  |  |  |
| miR-103 |  | 0.47 (0.26-0.67) |  | 0.46 [0.36;0.48] |  | 0.42 (0.19-0.66) | 0.38 [0.08;0.61] |
| miR-140 |  | -0.01 (-0.27-0.24) |  | -0.09 [-0.30;0.26] |  | 0.07 (-0.34-0.48) | -0.26 [-0.50;0.43] |
| miR-145 |  | 0.63 (0.4-0.86) |  | 1.24 [0.68;1.47] |  | 1.08 (0.6-1.56) | 0.49 [0.35;0.99] |
| miR-186 |  | 0.3 (0.13-0.47) |  | 0.52 [0.24;1.03] |  | 0.6 (0.25-0.94) | 0.26 [-0.02;0.55] |
| miR-21 |  | -1.38 (-1.5--1.26) |  | -1.29 [-1.60;-1.15] |  | -1.38 (-1.64-(-1.11)) | -1.43 [-1.62;-1.20] |
| miR-27a |  | -0.42 (-0.67-(-0.16)) |  | -0.29 [-0.55;0.04] |  | -0.17 (-0.74-0.41) | -0.47 [-0.86;-0.09] |
| miR-29a |  | 1.37 (1.2-1.54) |  | 1.45 [1.26;1.50] |  | 1.37 (1.22-1.53) | 1.36 [1.04;1.56] |
| miR-106a |  | -4.4 (-4.52-(-4.27)) |  | -4.43 [-4.60;-4.35] |  | -4.42 (-4.6-(-4.24)) | -4.44 [-4.60;-4.19] |

CI: Confidence Interval; IQR: Interquartile Range
